# CD122-targetted IL-2 signals cause acute and selective apoptosis of B cells in Peyer’s Patches

**DOI:** 10.1038/s41598-020-69632-5

**Published:** 2020-07-29

**Authors:** Ayushi Singh, Kunal Dhume, Joanne D. Tejero, Tara M. Strutt, K. Kai McKinstry

**Affiliations:** 1 Division of Immunity and Pathogenesis, Burnett School of Biomedical Sciences, College of Medicine, University of Central Florida, Orlando, FL 32827 USA; 2 NanoScience Technology Center, University of Central Florida, Orlando, USA

**Keywords:** Immunology, Cytokines, Lymphocytes, Mucosal immunology

## Abstract

Interleukin-2 (IL-2) has both pro- and anti-inflammatory properties that have been harnessed clinically and that are used experimentally to modulate leukocyte subsets in vivo. In mice, the bioavailability and half-life of IL-2 in vivo can be increased by complexing recombinant IL-2 with different clones of anti-IL-2 monoclonal antibodies that differentially target the cytokine to cells expressing different kinds of IL-2 receptors. While the impacts of systemic IL-2: anti-IL-2 antibody complex (IL-2C) administration are well-defined in the spleen and peripheral lymph nodes, how immune cells in the gut and gut-associated lymphoid tissues respond to IL-2C is not well characterized. Here, we analyze how major leukocyte populations in these tissues respond to IL-2C. We find that IL-2C targeting cells expressing IL-2 receptor beta cause an acute decrease in cellularity of Peyer’s Patches while cell numbers in the lamina propria and intraepithelial lymphocytes are unaffected. Cell contraction in Peyer’s Patches is associated with the apoptosis of multiple B cell subsets. Our results are important to consider for understanding off-target impacts of IL-2C regimes in experimental models and for considering how IL-2 may contribute to the etiology or severity of gut-associated conditions such as Crohn’s Disease.

## Introduction

Interleukin-2 is a pleiotropic cytokine that regulates responses of diverse cell types both during homeostasis and immune responses^1,2^. The ability of IL-2 to modulate these populations has led to its clinical use to treat cancer, by expanding NK cell and CD8 T cell populations within patients^3,4^, and to treat autoimmune conditions, largely by expanding FoxP3^+^ CD4^+^ regulatory T cells (Tregs)^5^. The immune-enhancing versus immune-dampening actions of IL-2 can be directed in vivo by targeting the cytokine to cells expressing distinct kinds of IL-2 receptors. One method of achieving such targeting is to create complexes of recombinant IL-2 and monoclonal anti-IL-2 antibodies that block sites on the cytokine recognized by either CD25 or CD122, the alpha and beta subunits of the IL-2 receptor, respectively^6^. In mice, the antibody clone S4B6 targets IL-2 to CD122^high^ cells, notably CD8 T cells and NK cells, while the clone JES6-1 clone targets IL-2 to CD25^high^ cells, typified by Tregs in the steady-state^7^. Such IL-2 complexes (IL-2C) also increase the half-life and bioavailability of IL-2 compared to delivery of soluble cytokine alone. IL-2C delivered systemically by intraperitoneal (i.p.) injection over the course of 3 consecutive days or longer has gained wide-spread use in diverse experimental models to activate or expand different kinds of immune cells. However, the off-target impacts of IL-2C administration in vivo are not fully described. A comprehensive understanding of how IL-2 signals impact leukocyte subsets at diverse tissue sites is required to minimize potential side effects and to properly interpret experimental findings.

One well-characterized side effect of high dose IL-2 treatment in cancer patients is the acute development of severe and acute gastrointestinal symptoms^8^. Mounting evidence also indicates that IL-2 may also have roles in the etiology and progression of intestinal inflammation associated with conditions like Crohn’s Disease (CD)^9–11^. A better understanding of how IL-2 signals affect leukocyte populations in the gut and gut-associated lymphoid tissues (GALT) under steady state conditions may thus provide insight into how the cytokine can impact disease states.

To address these knowledge gaps, we treated naive mice with IL-2C containing 2 µg of recombinant IL-2 for 3 consecutive days by i.p. injection. We have used this regime in previous studies centered on understanding how CD4 T cells form memory populations and protect against influenza virus^12–15^. We then evaluated impacts of IL-2C administration on major leukocyte subsets seen in the spleen and peripheral lymph nodes as well as those in the gut and GALT one day after the cessation of treatment.

Here, we show that CD122-targetted IL-2 signals delivered systemically by S4B6 IL-2C, while increasing cellularity of secondary lymphoid organs including mesenteric lymph nodes (mLN), do not significantly impact the numbers or distribution of leukocyte subsets within intraepithelial lymphocytes (IEL) in the gut or within the lamina propria (LP). In sharp contrast, S4B6 IL-2C unexpectedly induce a dramatic decrease in cell numbers within Peyer’s Patches (PP). The decrease in PP cellularity is caused by specific apoptosis of B cells that was not observed following JES6-1 IL-2C treatment, indicating that CD122-dependent IL-2 signals are responsible. We did not, however, observe changes indicative of direct IL-2-mediated signaling in PP B cells, in contrast to phenotypic impacts of S4B6 IL-2C on CD8 T cells that are well-known to directly respond to this IL-2C. Mechanistically, loss of PP B cells is associated with acute apoptosis in the absence of cellular division and is restricted mostly to B cells expressing IgM and/or IgD, spanning phenotypic distinctions of B-1 and B-2 subsets. Finally, we show that PP B cells recover within two weeks after cessation of IL-2C administration, indicating that the treatment does not permanently disrupt PP B cell homeostasis or the supporting PP architecture. These results may have relevance to the understanding of how IL-2 can impact gastrointestinal diseases and should be taken into account when interpreting results of mouse models employing S4B6 IL-2C.

## Results

### Systemic IL-2C administration reduces PP cellularity

To understand the extent to which systemic IL-2 signals have the capacity to impact immune cells in the gut and GALT in the steady state, we treated naive C57BL/6 mice for three consecutive days with i.p. injection with S4B6 IL-2C in PBS containing 2 µg of recombinant murine IL-2. We harvested tissues and analyzed major leukocyte subsets one day after the final IL-2C treatment and compared results to those obtained in control mice that were treated i.p. only with PBS for three days. We investigated the impacts of these IL-2 signals on the cellular composition of GALT and compared them to changes observed in the spleen and peripheral lymph nodes in the same animals. IL-2C-induced changes in systemic secondary lymphoid organs are well documented in the literature, including by our own studies^14^, and thus analyses of these tissues provides an internal control within individual mice to verify the efficacy of IL-2C treatment.

**Figure 1:**
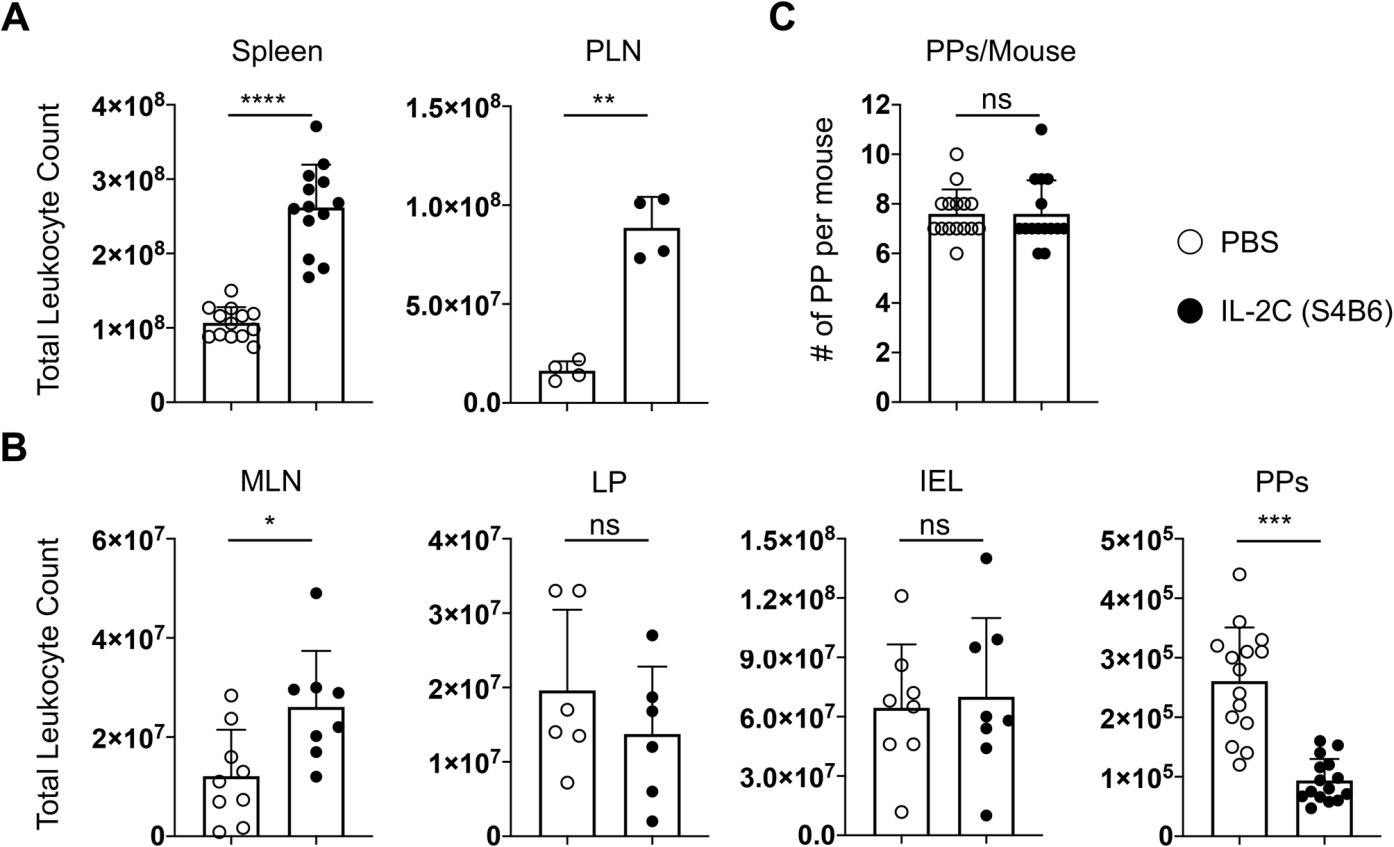
IL-2 signals increase cellularity of systemic secondary lymphoid organs but decrease PP cellularity. Naïve mice received S4B6 IL-2C or PBS alone for three consecutive days. On the fourth day, stated tissues were harvested and total cellularity was determined. (**A**) Summary of cell counts from spleen and peripheral lymph nodes (PLN) with each circle representing an individual control (open) or IL-2C-treated (closed) mouse. (**B**) Summary of cell counts for mesenteric lymph nodes (MLN), lamina propria (LP), intraepithelial lymphocytes (IEL) and Peyer’s Patches (PP). (**C**) The total number of PPs recovered per individual mouse. Data in A–C from 4 to 16 mice per group, with all error bars representing the standard deviation and significant differences determined with unpaired, two-tailed, students *t*-tests (*P < 0.05, **P < 0.005, ***P < 0.001, ****P < 0.0001, NS denotes no significant difference).

We first assessed the total cellularity of relevant tissues harvested from PBS- and IL-2C-treated mice. The cell count of the spleen and of pooled peripheral lymph nodes was increased ~ threefold after IL-2C treatment versus control mice (Fig. 1A), consistent with previous findings using a similar S4B6 IL-2C regime^16^. The cellularity of the mLN also increased after IL-2C treatment (Fig. 1B) but treatment did not impact the total number of mLN present (data not shown). There was no difference in treated or control mice in the number of cells found in the lamina propria or in the IEL compartment (Fig. 1B). Strikingly, the number of cells recovered from PPs following IL-2C treatment was reduced by at least twofold compared to control mice (Fig. 1B), but the number of individual PPs recovered from each mouse was not impacted by IL-2C administration (Fig. 1C). Together, these results demonstrate dramatically different impacts of S4B6 IL-2C administration on the cellularity of systemic versus mucosal secondary lymphoid organs, and little impact of IL-2 signals on cells in the lamina propria or on IELs.

### Selective loss of B cells in PP following S4B6 treatment

**Figure 2:**
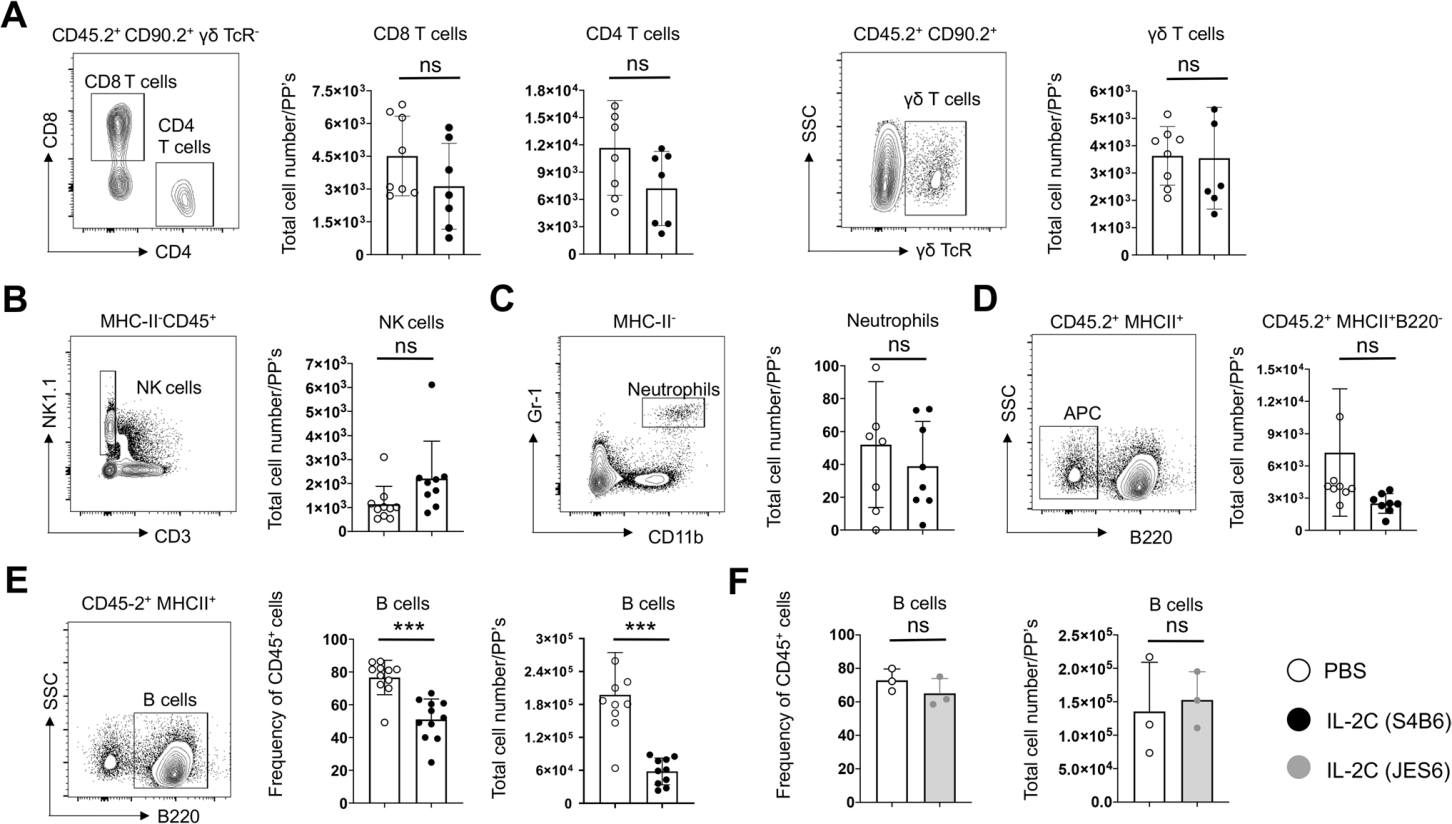
CD122-dependent IL-2 signals specifically reduce B cells in Peyer’s Patches. Representative flow cytometry staining and summary of total numbers of stated subsets of immune cells from individual mice treated with PBS alone (open circles) or S4B6 IL-2C (closed circles). (**A**) CD4, CD8 and γδ T cells, (**B**) NK cells, (**C**) neutrophils, and (**D**) antigen presenting cells. The gated populations presented in representative dot plots are indicated above each plot. (**E**) Representative staining, total number and frequency of B cells in PPs from individual control or S4B6 IL-2C-treated mice. (**F**) Total number and frequency of B cells in PPs of mice treated with PBS alone or with JES6-1 IL-2C (grey bars; one of two replicate experiments). All error bars represent the standard deviation and significant differences determined with unpaired, two-tailed, students *t*-tests (***P < 0.001, NS denotes no significant difference).

We next assessed major leukocyte populations by flow cytometry to determine the subset(s) of cells that are impacted in the PPs following systemic S4B6 IL-2C treatment. We saw no changes in the total numbers of CD4^+^, CD8^+^, or γδ^+^T cells comparing IL-2C-treated versus control mice (Fig. 2A). Similarly, IL-2C treatment did not impact the number of innate immune cells including NK cells (Fig. 2B), neutrophils (Fig. 2C), and bulk CD45^+^ MHC-II^+^ antigen presenting cells in PPs (Fig. 2D). In contrast, we did observe a significant decrease in the frequency and number of B cells within PPs identified by staining with CD45.2, MHC-II, and B220 (Fig. 2E). No changes in any of these populations were noted in the lamina propria or IELs (data not shown), which is consistent with observations shown in Fig. 1 of no overall changes in total cell numbers in these compartments.

These results are surprising as we, and others, have previously shown that this regime of S4B6 IL-2C administration drives dramatic increases in CD8 T cells and NK cells in the spleen, with smaller increases in CD4 and γδ T cells, and a modest increase in total B cells^14^. To determine whether the decrease in PP B cells required CD122-targeted IL-2 signals we next treated mice for 3 days with either PBS alone or with IL-2C made using the JES6-1 antibody clone, which targets IL-2 to CD25-expressing cells. In contrast to the effects of S4B6 IL-2C, JES6-1 IL-2C administration did not impact total PP cellularity (data not shown) or the number or frequency of B cells (Fig. 2F). JES6-1 IL-2C did, however, have a strong biological impact as the number of CD25^+^ FoxP3^+^ Tregs found in the spleen were increased by ~ 20 times, but no increase in Tregs was seen in PPs (data not shown). These observations indicate that the decrease in PP B cell numbers requires CD122-dependent IL-2 signals.

### S4B6 IL-2C cause apoptosis but not proliferation of PP B cells

**Figure 3:**
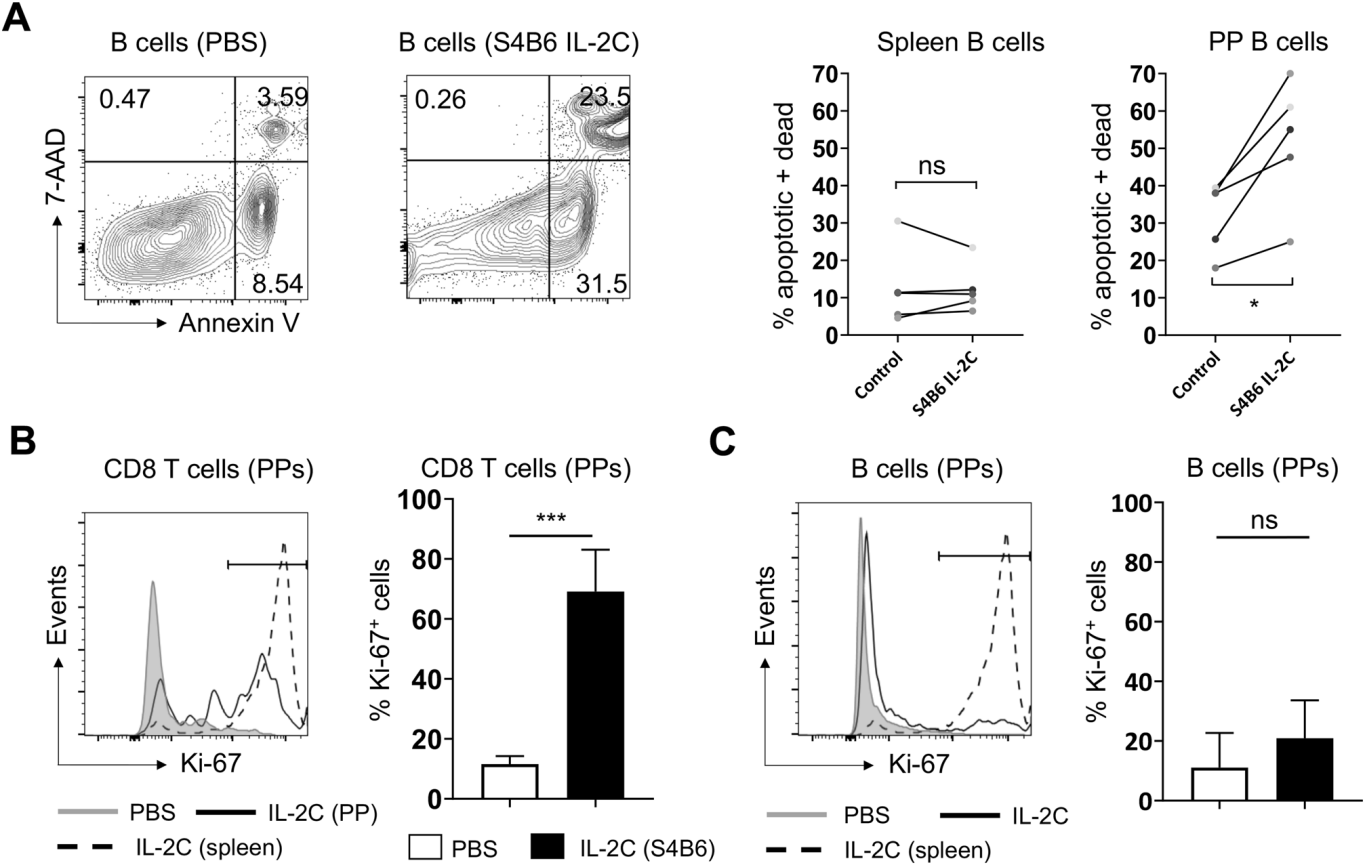
S4B6 IL-2C treatment causes apoptosis of PP but not splenic B cells. (**A**) Representative staining of Annexin V and 7-AAD on B cells in PP of control mice (left) and S4B6 IL-2C-treated mice (right), as well as summary analysis of the total frequency of dead and dying B cells from 5 mice per group (each connected set of data points represents one pair [control and S4B6-treated] of mice analyzed on the same day). (**B**) Representative staining and summary analysis from 5 mice per group of Ki67 staining in PP CD8 T cells in control (grey) and treated (black) mice, including the positive gate used in analysis, with splenic CD8 T cell Ki67 levels from treated mice as a control (dotted). (**C**) Similar analysis of Ki67 expression by PP B cells. All error bars represent the standard deviation and significant differences determined with unpaired, two-tailed, students *t*-tests, except for (A) where a paired two-tailed *t*-test was employed (*P < 0.05, ***P < 0.001, NS denotes no significant difference).

We next sought to identify the mechanism by which S4B6 IL-2C reduce B cell numbers in PPs. Changes to trafficking patterns associated with B cell migration during infection and following treatment of mice with CpG has been reported to decrease PP cellularity^17^. On the other hand, B cell apoptosis in PPs is also well-described during certain infections^18^ and following inflammation induced by sepsis^19^. To determine whether the IL-2C administration induces apoptosis of PP B cells, we compared staining with Annexin-5 and 7-AAD on B cells in the PPs versus B cells in the spleen. The frequency of total apoptotic (Annexin^+^7AAD^−^) and dead (Annexin^+^7AAD^+^) B cells was similar in spleens of control mice and mice that received S4B6 IL-2C. In contrast, dead and apoptotic B cells were significantly increased in IL-2C-treated PPs (Fig. 3A). Rates of apoptosis were higher in PP versus in the spleen even in control mice (Fig. 3A). The latter observation may reflect stresses associated with longer times needed to process GALT tissues for analysis versus for the spleen, and/or differences in homeostatic turnover. Apoptosis is often coupled in immune cells with their acute proliferation. IL-2C administration drove proliferation of CD8 T cells in PPs, as evidenced by Ki67 staining, but this was not as pronounced as the degree of Ki67 upregulation seen in CD8 T cells in the spleen (Fig. 3B). In contrast, the frequency of Ki67^+^ B cells was similarly low in control versus IL-2C-treated mice (Fig. 3C). Together, these results demonstrate that S4B6 IL-2C induce acute apoptosis of B cells within PP without induction of their proliferation, a hallmark functional response to IL-2.

### Direct IL-2 signaling to PP B cells by S4B6 IL-2C is not required to promote apoptosis

**Figure 4:**
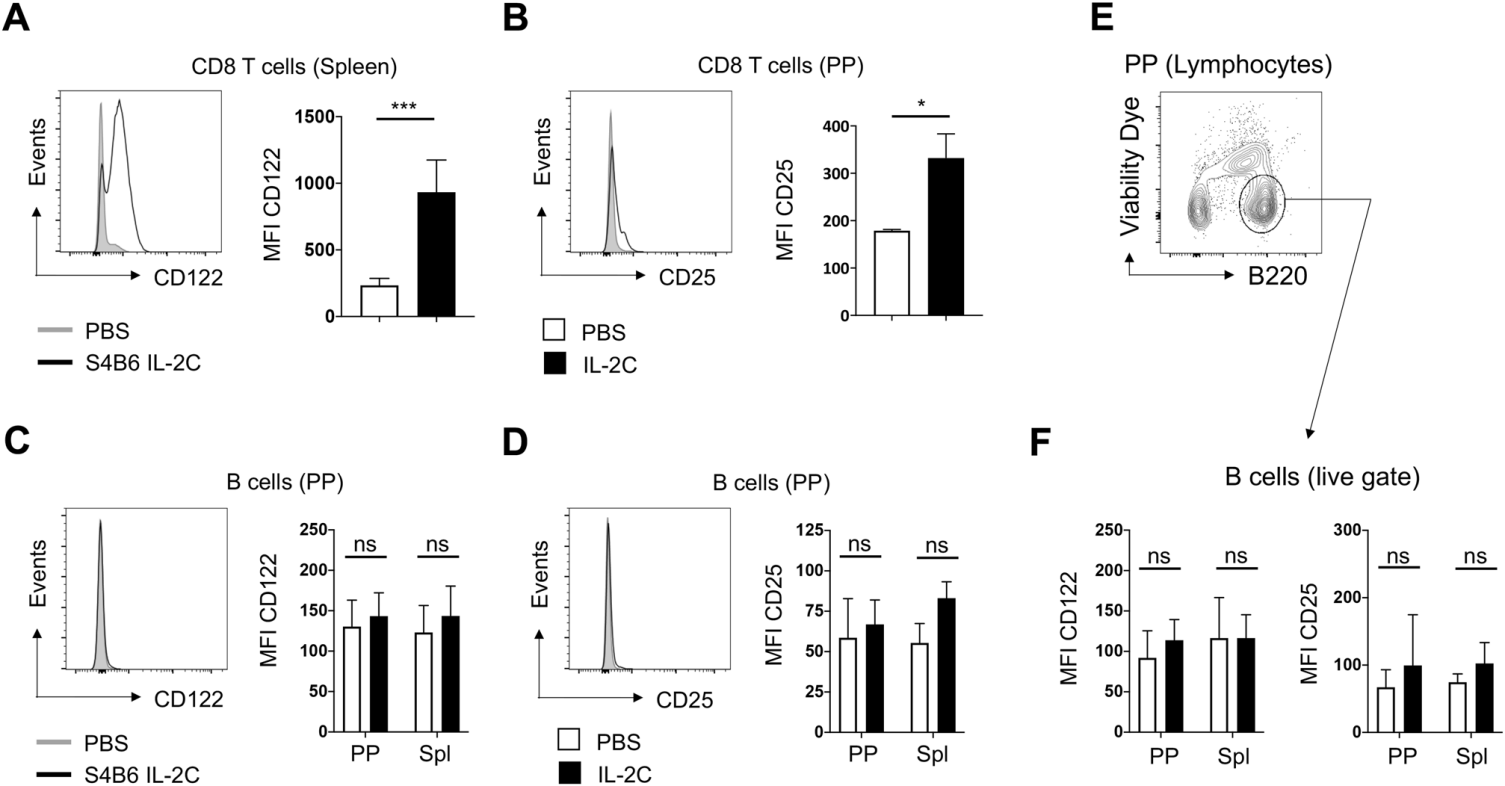
S4B6 IL-2C treatment does not impact IL-2 receptor expression on PP B cells. Representative staining and summary analysis of CD122 expression on CD8 T cells in control and IL-2C-treated mice in the (**A**) spleen and (**B**) PPs. Analysis of (**C**) CD122 and (**D**) CD25 expression on PP B cells. (**E**) Representative staining to identify viable B cells in PPs and (**F**) analysis of CD122 and CD25 expression on viable B cells from PPs and spleens isolated from IL-2C-treated and control mice. All data summarizes experiments with 4 mice per group. All error bars represent the standard deviation and significant differences determined with unpaired, two-tailed, students *t*-tests (*P < 0.05, ***P < 0.001, NS denotes no significant difference) in (**A**) and (**B**) and with ANOVA for (**C**), (**D**), and (**F**).

We next sought to determine whether the pro-apoptotic impact of S4B6 IL-2C on B cells in PP was associated with evidence of direct IL-2 signaling in the B cells. A well-known impact of IL-2 signaling is the acute upregulation of IL-2 receptor by cells receiving the signal^20^, and experimental evidence indicates that murine B cells can express functional IL-2 receptors^21–23^. Indeed, the proliferation of and increased number of CD8 T cells seen in the spleen, and less so in PPs, driven by S4B6 IL-2C correlated with significantly increased surface expression of CD122 (Fig. 4A,B) and a more modest increase in CD25 (data not shown). In contrast, CD122 (Fig. 4C) and CD25 (Fig. 4D) expressed by total B cells in PPs was not impacted by S4B6 IL-2C treatment. Splenic B cells, which did not undergo IL-2C-induced apoptosis, expressed levels of CD122 and CD25 that were similar to those on PP B cells and these were also not impacted by IL-2C treatment (Fig. 4C,D). To rule out the possibility that non-specific staining of dead or dying cells impacted the analysis above, we analyzed separate IL-2C-treated or control mice for CD122 and CD25 incorporating a viability dye to distinguish live B cells (Fig. 4E). Similar levels of both IL-2 receptor components were expressed by the live B cells in treated and control mice (Fig. 4F). Combined with the absence of PP B cell proliferation shown in Fig. 3, the lack of IL-2 receptor modulation following IL-2C treatment strongly indicates that PP B cell apoptosis induced by S4B6 IL-2C is not mediated by direct IL-2 signals to the B cells. These results also argue against the possibility that receipt of direct IL-2 signals by splenic but not PP B cells rescues the former but not the latter from apoptosis.

### Multiple B cell subsets are depleted in PP by S4B6 IL-2C

**Figure 5:**
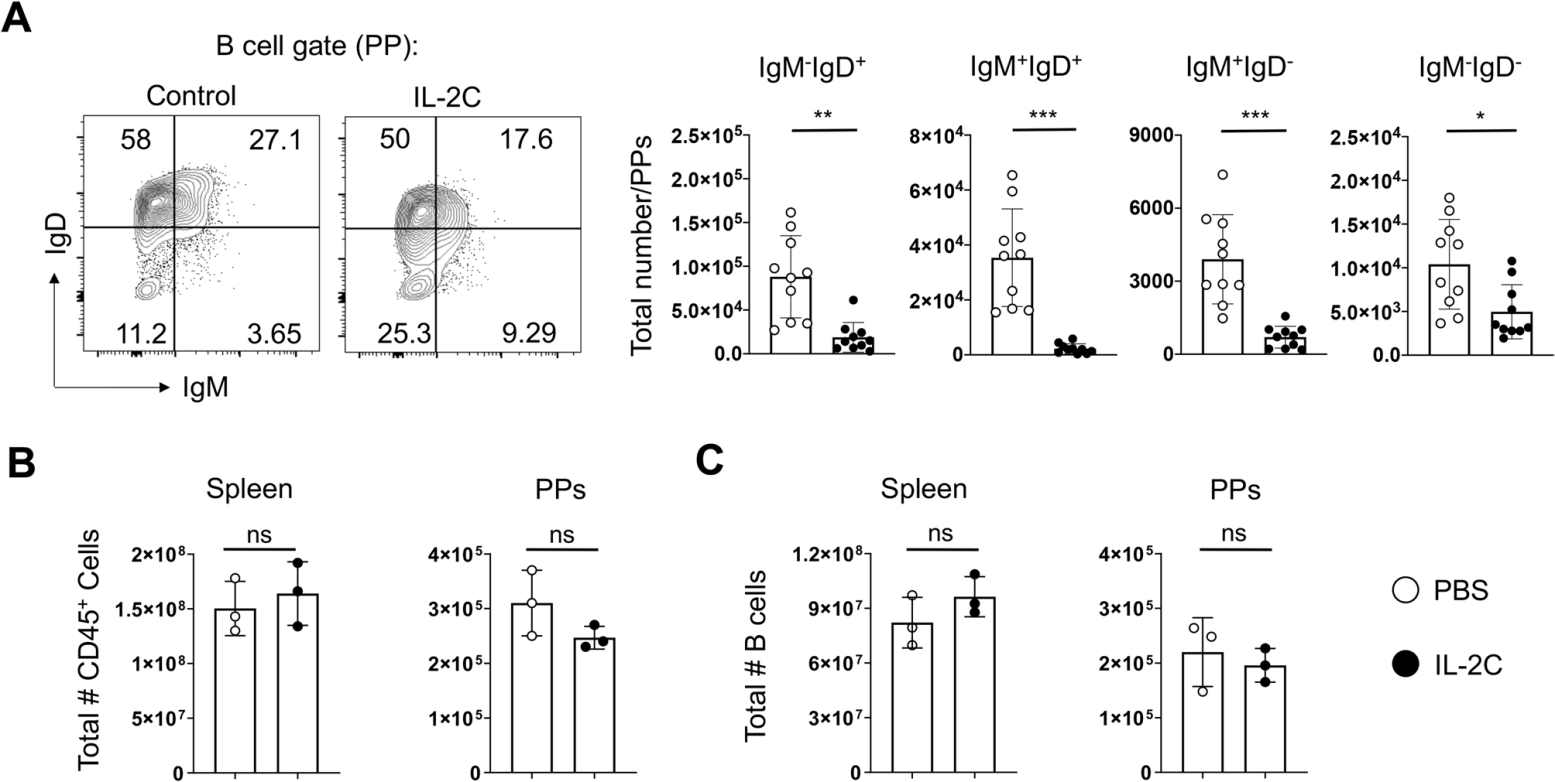
S4B6 IL-2C induce temporary loss of multiple B cell subsets in PPs. (**A**) Representative staining for IgD and IgM on B cells in PPs isolated from mice treated with either 3 consecutive treatments of S4B6 IL-2C or PBS. Summary analysis from 10 mice per group is shown for the number of B cells expressing the stated combinations of IgD and IgM in PPs. Mice were treated with S4B6 IL-2C or PBS for 3 days as in (**A**) but were analyzed 14 days after the final treatment. (**B**) The total number of CD45^+^ cells is shown for 3 mice per group in the spleen and PPs as well as (**C**) the total number of B cells in the spleen and PPs (*P < 0.05, **P < 0.005, NS denotes no significant difference).

We next sought to characterize what kind of B cells are most affected by IL-2C treatment in PPs, and whether the impact is permanent or transient. We thus isolated PPs from control or S4B6 IL-2C-treated mice and stained B cells to detect IgM and IgD expression. The majority of B cells in PPs isolated from control mice expressed either or both IgM and IgD, similar to previous findings^24^ (Fig. 5A). This was also the case in PP isolated from IL-2C-treated mice, but the frequency and total numbers of IgD and IgM positive cells, in all combinations, was significantly reduced compared to control mice (Fig. 5A). The majority of B cells in PPs are follicular (B-2) B cells that predominantly express high IgD and less IgM, with a much smaller population of B-1 B cells that express high IgM levels with little IgD^25^. Our analysis indicates similar declines (about fourfold) in the absolute number of both B-1 and B-2 B cells in PP using these criteria (Fig. 5A). IgM^+^IgD^+^ B cells, which may represent a specialized subset of precursor cells for IgA^+^ plasma cells in the lamina propria^24^, were also reduced by a similar degree by IL-2C treatment (Fig. 5A). In contrast, the total number of B220^+^ cells that did not express IgM or IgD was only decreased only by about one half by IL-2C treatment, and in some experiments this subset was not reduced significantly (Fig. 5A).

To ask if the IL-2 signals result in long-term or permanent changes in B cell populations in PPs, we treated mice for 3 days with IL-2C and waited 14 days before analysis. In contrast to analysis performed one day after the final IL-2C treatment, we observed no differences in either the total cellularity of PPs in treated versus control mice (Fig. 5B) or the total number of B cells present (Fig. 5C), and the IgM^+^/IgD^+^ B cell profiles were not different between groups (data not shown). These results indicate that the IL-2C-induced alterations in the cellular composition of PPs are not permanent and that PP B cells return to baseline levels within 14 days after delivery of exogenous IL-2 signals are stopped.

## Discussion

IL-2’s ability to act as both a pro- and anti-inflammatory cytokine offers many novel applications for its use clinically and experimentally. These attributes, however, also create the possibilities of off-target impacts as well as severe side-effects to be associated with IL-2 treatment regimes. A detailed mechanistic understanding of how immune cells residing in different tissues are impacted by different kinds of IL-2 signals is thus required. As part of our previous studies, we delivered S4B6 IL-2C systemically and investigated its impacts on immune cells in the spleen and lungs. We found, overall, that the general patterns observed in the spleen following S4B6 IL-2C administration were also seen in the lung, with increases in numbers of T cells and especially NK cells^14^. These results indicated that the regulation by IL-2 of major populations of immune cells present in secondary lymphoid organs and at mucosal sites is similar. Our results presented here, however, reveal that the same S4B6 IL-2C signals have a very different impact on shaping the landscape of immune cells present in the gut, and even between different GALT compartments. Further studies are required to determine the extent to which the different responses observed in the lung and the gut, and potentially other mucosal tissues, are due to unique environmental conditions present versus unique gene expression and/or epigenetic regulation by leukocytes within different tissues.

Our observations of IL-2C-induced reductions in total cellularity in PPs, but not in other GALT or in secondary lymphoid organs, are surprising and novel. They may offer mechanistic insight to better understand the onset and severity of many gastrointestinal disease states. For example, there is evidence from human studies to support that PPs may be intimately involved in the etiology of CD^26,27^. Decreased PP cellularity and B cell apoptosis has also been observed prior to symptoms of disease in colitis^28^. Furthermore, the disruption of PPs has been linked to more severe symptoms in a mouse model of DSS-induced colitis^29^. These and other findings indicate that perturbations in PP homeostasis may be a critical event in either disease establishment or disease progression. The mechanisms responsible for PP disruption affecting disease progression are complex and may not be uniform across all situations. One possibility supported by our observations is that IL-2 signals may impact IgA production in the gut, as IgM^+^/IgD^+^ B cells have been shown to be precursors for IgA^+^ producing cells in the lamina propria^24^. As this subset was reduced most strikingly by S4B6 IL-2C treatment in our studies, it is possible that strong IL-2 signals compromise IgA-dependent protective or regulatory mechanisms associated with induction of disease states^30^. We point out, however, that the short-term experiments summarized here do not allow for an analysis of whether and how IL-2C-dependent changes in B cells ultimately impact antibody production in the gut. A separate body of evidence supports that a reduction or ablation of B cell numbers, by treatment with anti-CD20 B cell-depleting antibody, for example, can aggravate IBD symptoms in humans^31–33^ and in mice^34^. This highlights the broad ability of B cells to modulate intestinal bowel disease. Further studies using IL-2C treatment, as in the work presented here, in genetic or induced models of IBD in mice may provide novel insight into how the perturbation of PPs and B cell subsets within them can impact disease progression and severity.

We did not see any of the most common phenotypic indications of direct IL-2 signaling in PP B cells that are seen in T cells stimulated by IL-2 including an increase in forward scatter, increases in Ki67 levels, and changes in expression of components of the IL-2 receptor. Similar findings of acute B cell, but not T cell, death in PPs have been observed in a model of sepsis in mice^19^. The apoptotic impact on B cells during sepsis was found not to be caused by direct endotoxin-dependent signals to B cells. Sepsis is characterized by an acute systemic inflammatory response, and we found that S4B6 IL-2C injection, as used in the studies presented here, similarly induces robust and systemic inflammation^14^. Combined, these observations suggest that key signals regulating B cell apoptosis in both our studies and those of Ayala and colleagues induced during sepsis are most likely related to either the upregulation and/or downregulation of soluble factors associated with the ‘cytokine storm’ induced by the respective treatments. Further studies are required to determine the mechanisms involved and to identify the specific factor or factors that are critical for inducing the selective apoptotic response in PP B cells presented here. Interestingly, we recently found that the inflammatory responses detected in the serum of mice induced by systemic S4B6 and JES6-1 IL-2C administration have both overlapping and unique constituents in terms of the individual cytokines and chemokines detected^15^. Given that S4B6 IL-2C caused B cell apoptosis in PPs but JES6-1 IL-2C did not, these findings may provide a short-list of candidate targets, unique to S4B6 IL-2C-induced inflammation spectrum, that may be involved in potentiating PP B cell apoptosis. However, given that systemic inflammation induced by IL-2 in mice and humans^35^ is extremely broad, we point out that it is unlikely that changes in the levels of any single inflammatory factor are responsible for the impact on PPs that we describe.

Our analysis also indicates that the cellular sources of inflammation most impacted by S4B6 versus JES6 IL-2C administration may be responsible for altering the PP B cell landscape. Fox example, NK cells are dramatically expanded by the S4B6 IL-2C, but not by the same dose and treatment schedule of JES6-1 IL-2C^15^. NK cells, but not CD8 T cells, are required for vascular leak syndrome induced by high dose IL-2 administration in mice^36^, mirroring a major toxicity of IL-2 treatment seen in humans^4^. We also found that NK cells, but not T cells, expanded by IL-2 drive inflammatory responses that worsen the outcome of influenza infection in mice^14^. We did not, however, see significantly increased NK cells in PPs of mice treated with S4B6 IL-2C. In fact, no changes in major subsets of innate or adaptive cells in PPs were seen after S4B6 IL-2C treatment other than in the B cell compartment. This suggests that the source(s) of factors responsible for initiating B cell apoptosis in PPs are systemic rather than local. Indeed, previous studies found that systemic administration of type I IFN or polyI:C to mice rapidly disrupted PPs^37^. In contrast to our observations, these treatments resulted in the loss of both B and T cells and reduced the total number of PPs present in mice. PP cellularity, however, quickly recovered to control levels after cessation of type I IFN signaling^37^, similar to the rebound in PP B cell numbers we describe after cessation of IL-2C administration. Determining the fine kinetics of PP reconstitution after IL-2-induced B contraction could provide insight into mechanisms regulating PP dynamics in infectious versus non-infectious disease states.

It is also possible that stromal cells in PPs respond to the IL-2C and that processes initiated by these cells result in B cell death. Indeed, endothelial cells in the lung express functional IL-2 receptors and contribute to edema following IL-2 administration^38^. It is important to stress that stromal versus haemopoietic sources of IL-2-dependent signals required for B cell apoptosis in PPs are not mutually exclusive possibilities. Further experiments are required to delineate those cells responding to IL-2 that are responsible for the impacts on PPs described here.

The apoptotic versus non-apoptotic impact of IL-2C treatment on B cells present in the spleen versus PP, respectively, may also reflect intrinsic differences in the way that the B cells in these tissues are able to respond to inflammatory signals. This hypothesis is supported by studies from Burger and Vitetta finding that B cells in the spleen, peripheral lymph nodes, and PPs display markedly different responses to stimuli such as LPS and IL-4^39^. A non-mutually exclusive possibility is that other cellular populations within PP and other tissues intrinsically respond differently to inflammatory signals induced by IL-2C to indirectly modulate B cell fate. For example, studies have found that splenic versus PP dendritic cells have different capacities to produce cytokines like IL-10 and IL-12 upon stimulation^40^. A comprehensive description of how cytokines like IL-2 affect distinct leukocyte subsets within individual tissues, whether directly or indirectly, may provide novel therapeutic applications or inform on development of countermeasures to reduce side effects associated with treatments using IL-2.

In summary, our results indicate that systemically administered IL-2C differentially impact leukocytes in the GALT versus in systemic secondary lymphoid organs like the spleen and peripheral lymph nodes. While cells in the lamina propria and IELs are largely unaffected, PPs are markedly impacted by short-term IL-2C treatment due to the induction of apoptosis in multiple subsets of B cells. Given the widespread use of S4B6 IL-2C in mouse experiments, it is important to consider how this unexpected consequence of IL-2C treatment might affect outcomes, especially in studies focused on responses in GALT. This work may also lead to greater insight into how inflammatory signals act at different mucosal sites, with implications for understanding the etiology of conditions such as Crohn’s Disease.

## Materials and methods

### Mice

All experimental animal procedures were conducted in accordance with the University of Central Florida’s Institutional Animal Care and Use Committee guidelines. Age-matched female C57BL/6J mice between 6 and 10 weeks of age were used in all experiments. The mice were originally obtained from Jackson Laboratories and were bred at the University of Central Florida Vivarium at Lake Nona.

This study was compliant with all of the ethical regulations applicable to animal research and was reviewed and approved by the University of Central Florida Institutional Animal Care and Use Committee.

### IL-2 complex treatment

Mice were treated for 3 consecutive days by i.p. injection of IL-2 complexes (IL-2C) in 200 μL of PBS. The IL-2C were prepared as previously described^12,14^ by premixing 2 μg of recombinant murine IL-2 (ThermoFisher) with 20 μg of the anti-mouse IL-2 antibody clone S4B6-1 (BD Biosciences). In some experiments IL-2C were also made using the anti-IL-2 antibody clone JES6-1A12 (ThermoFisher). The IL-2C were incubated at room temperature for 20 min before i.p. injection. Control mice were injected with 200 μL of PBS alone.

### Tissue harvest and processing

One day following the final IL-2C treatment, experimental and control mice were euthanized and the spleen, peripheral lymph nodes, and the intestine along with the fat and mesentery were harvested. All organs except the intestine were harvested into RPMI 1640 media containing 2 mM L-Glutamine, 100 IU penicillin, 100 μg/mL streptomycin, 10 mM Hepes, 50 μM 2-mercaptoethanol, and 7.5% fetal bovine serum. The organs were each made into single cell suspensions by mechanical disruption and passage through a 100 μm steel mesh screen.

The intestines were harvested into PBS and further processed as follows: mesenteric lymph nodes were carefully excised followed by removal of visible PPs from the intestine in a dark room. Single cell suspensions were prepared by mechanical disruption and the cells were then filtered through a 100 μm cell strainer (Fisher). The intestinal tissue was then flushed with ice cold PBS, cut longitudinally, and washed thoroughly to remove lumen contents. The tissue was then cut into ~ 0.5 cm pieces and processed according to methods specified by the lamina propria dissociation kit, using a gentleMACS tissue dissociator with heaters (Miltenyi), to yield single cell suspensions of lamina propria cells as well as IELs. The cells were washed thoroughly and resuspended in RPMI 1640 media supplemented as described above. Cell counts were determined with a hemocytometer and trypan blue exclusion using light microscope.

### Flow cytometry

Cells were stained for flow cytometry analysis as previously described^41^. Briefly, cell suspensions were washed, resuspended in flow cytometry staining buffer (PBS plus 0.5% bovine serum albumin and 0.02% sodium azide), and then incubated on ice (2–8 °C) for 30 min with minimal exposure to light. The samples were treated with anti-FcR (2.4G2, BioXcell) to block non-specific binding. Saturating concentrations of the fluorochrome-labeled antibodies were added for surface staining: anti-CD45.2 (104(RUO)), anti-CD90.2 + (53–2.1), anti-CD4 (GR 1.5), anti-CD8 (53–6.7), anti-γδ (GL3), anti-MHCII (M5/114.15.2), anti-CD11c (N418), anti-CD11b (M1/70), anti-B220 (RA3-682), anti-IGM (RMM-1), anti-IGD (11-26c.2a), anti-CD3 (71A2), anti-NK1.1 (553165), anti-Gr-1 (RB6-8C5), anti-CD25 (7D4), anti-CD122 (TMb-1(RUO). Viability dye staining was performed according to manufacturer’s instructions using the Zombie Violet fixable viability kit (Biolegend). All antibodies were purchased from BD Pharmigen, ThermoFisher, or Biolegend.

Nuclear staining for Ki67 was performed according to the eBioscience Transcription Factor Staining Buffer Set (ThermoFisher) after completion of relevant surface staining (as described above). Levels of Ki67 were detected using anti-Ki67 antibody (SolA15) from ThermoFisher. Apoptosis and cell death were measured by flow cytometry using Annexin V Apoptosis Detection Kit with 7-AAD as per manufacturer’s instructions (BioLegend) after relevant surface staining as described above.

All samples were fixed in 1% paraformaldehyde and run on the same day using a FACS Canto flow cytometer (BD Biosciences). All analysis of flow cytometry data was done using FlowJo (Tree Star) software.

### Statistical analysis

All statistics were analyzed by GraphPad Prism. Unpaired two-tailed student t tests were used to determine the significance by which two normally distributed groups differed unless otherwise stated in the figure legends. A *P* value of < 0.05 was deemed significant. All error bars represent the standard deviation. In all figures, significance is indicated as *P < 0.05, **P < 0.005, and ***P < 0.0005.
